# Supplementary material for: P-TRAP: a Panicle Trait Phenotyping tool
Source: BMC Plant Biol. 2013 Aug 29;13:122. doi: 10.1186/1471-2229-13-122 (PMC3848748; doi:10.1186/1471-2229-13-122)
Supplement: Additional file 7 — P-TRAP source code. The Netbeans project that contains the source code of the application. [file 1471-2229-13-122-S7.zip › javadoc.zip!/javadoc/org-bioalg-imageprocessor/org/bioalg/imageprocessor/Kernels.html]

Kernels (Image Processor)


JavaScript is disabled on your browser.


- Package
- Class
- Tree
- Deprecated
- Index
- Help

*org.bioalg.imageprocessor 1.0*

- Prev Class
- Next Class

- Frames
- No Frames

- All Classes

- Summary:
- Nested |
- Field |
- Constr |
- Method

- Detail:
- Field |
- Constr |
- Method


org.bioalg.imageprocessor

## Class Kernels

- java.lang.Object
- - org.bioalg.imageprocessor.Kernels

- ---

    

  ```
  public class Kernels
  extends java.lang.Object
  ```

  Several kernels created by this class. They are useful for Math morphology
  and some other image based operations.

  Author:
  :   aanjos and faroq

- - ### Field Summary

    Fields

    | Modifier and Type | Field and Description |
    | --- | --- |
    | `static double[][]` | `horizontalLinesKernel` |
    | `static double[][]` | `verticalLinesKernel` |
  - ### Constructor Summary

    Constructors

    Constructor and Description

    `Kernels()`
  - ### Method Summary

    Methods

    | Modifier and Type | Method and Description |
    | --- | --- |
    | `static int[][]` | `generateCross(int r)` |
    | `static int[][]` | `generatedagger(int r)` |
    | `static java.util.ArrayList<java.awt.Point>` | `generateDiskAsPoints(int r, java.awt.Point center)` generate a disk mask for math morphology |
    | `static int[][]` | `generateDiskKernel(int r)` generate a disk mask for math morphology |
    | `static int[][]` | `generateEllipseKernel(int r1, int r2)` generate an ellipse mask for math morphology |
    | `static int[][]` | `generateemptyDiskKernel(int r)` |
    | `static int[][]` | `generateframe(int r)` |
    | `static int[][]` | `generateframe(int r, int c)` |
    | `static java.util.ArrayList<java.awt.Point>` | `generateframe(int r, int c, java.awt.Point center)` |
    | `static java.util.ArrayList<java.awt.Point>` | `generateframe(int r, java.awt.Point center)` |
    | `static int[][]` | `generateInvertedDiskKernel(int r)` |
    | `static int[][]` | `generateSqureKernel(int r)` |
    | `static double[][]` | `getFullGaussian(double sigma)` |
    | `static double[][]` | `getFullLoG(double sigma)` |
    | `static void` | `normalizeKernel(double[][] k)` |
    | `static void` | `printintKernel(int[][] k)` |
    | `static void` | `printKernel(double[][] k)` |
    | `static void` | `printpointKernel(java.util.ArrayList<java.awt.Point> k, int radius)` |
    | `static int[][]` | `reflect(int[][] kernel)` used in eroding a binary image using a dilate code |
    | `static double[][]` | `sharpen()` |

    - ### Methods inherited from class java.lang.Object

      `clone, equals, finalize, getClass, hashCode, notify, notifyAll, toString, wait, wait, wait`

- - ### Field Detail


    - #### horizontalLinesKernel

      ```
      public static double[][] horizontalLinesKernel
      ```


    - #### verticalLinesKernel

      ```
      public static double[][] verticalLinesKernel
      ```
  - ### Constructor Detail


    - #### Kernels

      ```
      public Kernels()
      ```
  - ### Method Detail


    - #### sharpen

      ```
      public static double[][] sharpen()
      ```


    - #### getFullGaussian

      ```
      public static double[][] getFullGaussian(double sigma)
      ```


    - #### getFullLoG

      ```
      public static double[][] getFullLoG(double sigma)
      ```


    - #### normalizeKernel

      ```
      public static void normalizeKernel(double[][] k)
      ```


    - #### printKernel

      ```
      public static void printKernel(double[][] k)
      ```


    - #### printintKernel

      ```
      public static void printintKernel(int[][] k)
      ```


    - #### printpointKernel

      ```
      public static void printpointKernel(java.util.ArrayList<java.awt.Point> k,
                          int radius)
      ```


    - #### reflect

      ```
      public static int[][] reflect(int[][] kernel)
      ```

      used in eroding a binary image using a dilate code


    - #### generateDiskKernel

      ```
      public static int[][] generateDiskKernel(int r)
      ```

      generate a disk mask for math morphology

      Parameters:
      :   `radius` -

      Returns:


    - #### generateDiskAsPoints

      ```
      public static java.util.ArrayList<java.awt.Point> generateDiskAsPoints(int r,
                                                             java.awt.Point center)
      ```

      generate a disk mask for math morphology

      Parameters:
      :   `radius` -

      Returns:


    - #### generateemptyDiskKernel

      ```
      public static int[][] generateemptyDiskKernel(int r)
      ```


    - #### generateInvertedDiskKernel

      ```
      public static int[][] generateInvertedDiskKernel(int r)
      ```


    - #### generateEllipseKernel

      ```
      public static int[][] generateEllipseKernel(int r1,
                                  int r2)
      ```

      generate an ellipse mask for math morphology

      Parameters:
      :   `r1` -
      :   `r2` -

      Returns:


    - #### generateSqureKernel

      ```
      public static int[][] generateSqureKernel(int r)
      ```


    - #### generateframe

      ```
      public static int[][] generateframe(int r)
      ```


    - #### generateframe

      ```
      public static int[][] generateframe(int r,
                          int c)
      ```


    - #### generateframe

      ```
      public static java.util.ArrayList<java.awt.Point> generateframe(int r,
                                                      java.awt.Point center)
      ```


    - #### generateframe

      ```
      public static java.util.ArrayList<java.awt.Point> generateframe(int r,
                                                      int c,
                                                      java.awt.Point center)
      ```


    - #### generateCross

      ```
      public static int[][] generateCross(int r)
      ```


    - #### generatedagger

      ```
      public static int[][] generatedagger(int r)
      ```


- Package
- Class
- Tree
- Deprecated
- Index
- Help

*org.bioalg.imageprocessor 1.0*

- Prev Class
- Next Class

- Frames
- No Frames

- All Classes

- Summary:
- Nested |
- Field |
- Constr |
- Method

- Detail:
- Field |
- Constr |
- Method
